# Integrative genomic analyses of APOBEC-mutational signature, expression and germline deletion of *APOBEC3* genes, and immunogenicity in multiple cancer types

**DOI:** 10.1186/s12920-019-0579-3

**Published:** 2019-09-18

**Authors:** Zhishan Chen, Wanqing Wen, Jiandong Bao, Krystle L. Kuhs, Qiuyin Cai, Jirong Long, Xiao-ou Shu, Wei Zheng, Xingyi Guo

**Affiliations:** Division of Epidemiology, Department of Medicine, Vanderbilt Epidemiology Center, Vanderbilt-Ingram Cancer Center, Vanderbilt University Medical Center, Nashville, TN 37203 USA

**Keywords:** Pan-cancer, APOBEC, Gene expression, Isoforms, APOBEC-signature mutations, Germline APOBEC3A/B deletion, Neoantigen, TILs

## Abstract

**Background:**

Although APOBEC-mutational signature is found in tumor tissues of multiple cancers, how a common germline *APOBEC3A/B* deletion affects the mutational signature remains unclear.

**Methods:**

Using data from 10 cancer types generated as part of TCGA, we performed integrative genomic and association analyses to assess inter-relationship of expressions for isoforms *APOBEC3A* and *APOBEC3B*, APOBEC-mutational signature, germline *APOBEC3A/B* deletions, neoantigen loads, and tumor infiltration lymphocytes (TILs).

**Results:**

We found that expression level of the isoform uc011aoc transcribed from the *APOBEC3A/B* chimera was associated with a greater burden of APOBEC-mutational signature only in breast cancer, while germline *APOBEC3A/B* deletion led to an increased expression level of uc011aoc in multiple cancer types. Furthermore, we found that the deletion was associated with elevated APOBEC-mutational signature, neoantigen loads and relative composition of T cells (CD8+) in TILs only in breast cancer. Additionally, we also found that APOBEC-mutational signature significantly contributed to neoantigen loads and certain immune cell abundances in TILs across cancer types.

**Conclusions:**

These findings reveal new insights into understanding the genetic, biological and immunological mechanisms through which *APOBEC* genes may be involved in carcinogenesis, and provide potential genetic biomarker for the development of disease prevention and cancer immunotherapy.

## Background

Somatic mutations are one of the most common causes of carcinogenesis. Recent studies have revealed that somatic mutations can be characterized by several distinct patterns, termed mutational signature. A particular signature mutation has been found to be driven by a sub-family of the human APOBEC (apolipoprotein B mRNA editing enzyme, catalytic polypeptide-like) gene. Kataegis, a phenomenon described cluster mutation along genome, are associated with APOBEC enzymatic activity [1]. The APOBEC-mediated mutagenesis, or mutational signature, substantially contribute to the overall mutation burden in the spectrum of human cancers, especially in bladder and breast cancers [2–12]. Two members of the gene family, *APOBEC3A* and *APOBEC3B*, have been known to play a role in inducing APOBEC-mutational signature [4, 9, 11, 13]. The APOBEC cytidine deaminase C, within the TCW trinucleotide motif that frequently changes to T or G mutations, have been ubiquitously observed in human cancer [1, 9]. A common germline deletion covering the last intronic of *APOBEC3A* to the last exon of *APOBEC3B* (called *APOBEC3A/B)* is found to increase APOBEC-mutational signature in breast cancer, as a similar pattern driven by the elevated *APOBEC3A* or *APOBEC3B* expressions [3]. A further study has shown that a fusion protein that is generated by germline *APOBEC3A/B* deletion has a higher expression level than the APOBEC3A protein [2]. Previous genome-wide association studies (GWAS) have shown that germline *APOBEC3A/B* deletion is not associated with breast cancer risk in several studies in European populations [14–18]. However, the association of the deletion with breast cancer risk in European populations has been observed in other studies [19, 20]. In our recent work, using the GWAS identified single nucleotide polymorphisms (SNPs) from The Breast Cancer Association Consortium (BCAC) [21], we found that a risk allele of the GWAS-identified SNP rs12628403 is significantly associated with a decreased expression of the *APOBEC3B* gene, supporting that germline *APOBEC3A/B* deletion contributes to breast cancer risk in European populations [22]. In Asian populations, all previous studies, together with our own work, have shown that this germline *APOBEC3A/B* deletion increased breast cancer risk [23–25]. In contrast to the findings in breast cancer, the germline *APOBEC3A/B* deletion was not observed in other cancer types with known enriched APOBEC-mutational signature, such as bladder cancer [11]. The underlying mechanism for why the specific association occurs only with breast cancer remains unclear.

It is known that neoantigens (or neoepitopes) arise from missense somatic mutations in cancer cells [26]. Neoantigens presented on the cell surface in the context of a major histocompatibility complex (MHC) of tumor tissues could be recognized by T cells as foreign antigens [27]. In a tumor microenvironment, a significant proportion of Tumor-infiltrating lymphocytes (TILs) that are comprised of immune cells, primarily from CD8+ cytotoxic T-cells (CTLs), has been observed in many cancer types, including breast cancer [28]. Thus, we hypothesized that APOBEC-mutational signature may affect cancer immunogenic abilities, such as attracting immune cells in TILs, which is likely mediated by affecting neoantigens. Recent studies have investigated the differentially-expressed genes between samples that were predicted to carry germline *APOBEC3A/B* deletion and samples that were predicted to have no deletion. The studies have shown that, based on gene set enrichment analyses, these differentially expressed genes significantly enriched the function of immune activation [25, 29]. Another study recently showed that APOBEC mutational signature was significantly correlated with immunotherapy response in non-small cell lung cancer [30]. However, the influence of APOBEC-mutational signature and germline *APOBEC3A/B* deletion on neoantigens and TILs remains largely unexplored, especially in pan-cancer studies.

The Cancer Genome Atlas (TCGA) has generated massive quantities of high-dimensional genetic and genomic data, including whole genome and exome sequencing, array-based genotypes, and RNA sequencing data, for many cancer types. The data resource provides an unprecedented opportunity to investigate APOBEC-mutational signature and immunogenicity in relation to *APOBEC* expression and germline *APOBEC3A/B* deletion. In particular, how APOBEC-mutational signature are affected by the individual isoforms of *APOBEC3A* and *APOBEC3B* still remain largely unexplored. Especially, the analysis challenge of the complexity of alterative splicing has been related to the isoform uc011aoc, transcribed from an *APOBEC3A/B* chimera that is primarily generated by germline *APOBEC3A/B* deletion. In this study, using data generated from approximately 4000 tumor samples across 10 cancer types from TCGA, we performed integrative genomic and association analyses of the isoform expressions of *APOBEC3A* and *APOBEC3B*, APOBEC-mutational signature, germline *APOBEC3A/B* deletion, neoantigen loads and TILs.

## Methods

### Data resources

This study utilized the sample resources from TCGA, including a total of 3937 samples with gene expression and somatic mutations data generated for 10 cancer types: bladder (*N* = 388), breast (*N* = 961), cervical (*N* = 185), lung adenocarcinoma (*N* = 475), lung squamous carcinoma (*N* = 178), head and neck (*N* = 498), stomach (*N* = 368), pancreas (*N* = 119), thyroid (*N* = 485) and kidney (*N* = 280). We included these cancer types for the investigation as they are known to have a significant enrichment of APOBEC-mutational signature [1].

### The measurement of isoform expression for *APOBEC3A* and *APOBEC3B*

We downloaded the human gene annotation from the Table Browser of the UCSC genome browser (https://genome.ucsc.edu/cgi-bin/hgTables). The six isoforms were investigated, including uc003awn, uc011aob, uc011aoc, uc003awo, uc003awp and uc003awq, transcribed from the genes APOBEC3A and APOBEC3B. The normalized expression levels for each isoform in tumor tissue samples have been measured using RNA-Seq by Expectation Maximization (RSEM) by the group of GDAC. A log2 transform of the RSEM values was applied to fit a better distribution for the downstream analysis.

### The data processing of APOBEC-mutational signature

The APOBEC-mutational signature was measured by the total count of TCWs changing to either TTW or TGW mutations from the signature mutation analysis by GDAC [1, 9]. The proportion of APOBEC-mutational signature relative to total mutations was measured as “[tCw_to_G + tCw_to_T]_per_mut”, in the column marked “Mutsig_maf_modified.maf_sorted_sum_all_fisher_Pcorr.txt” in the file. The number of total mutations for each sample was also extracted from the column “mutations” in the file. The number of APOBEC-mutational signature was also extracted from the column “tCw_to_G + tCw_to_T”. We applied a log2 transform to the number of APOBEC-mutational signature to fit a better distribution for the downstream analysis.

### The identification of germline *APOBEC3A/B* deletion

We downloaded the germline deletion data for the samples in TCGA using the whole genome sequencing and whole exome sequencing data from a previous study [3]. The germline deletions for additional samples that did not have sequencing data were identified using array-based genotype data. In our recent deep whole genome sequencing project involving breast cancer, we identified a ~ 29 kb common germline deletion covering from the last exon of the *APOBEC3A* gene to the last exon of the *APOBEC3B* gene [31]. The deletion breakpoint was localized on chromosome 22 between 39,358,340 and 39,388,452 (hg19). Using the coordinates of the deletion breakpoint as a reference, we examined the segmented copy number data within the region to determine germline *APOBEC3A/B* deletion for samples. A median value of segmented copy number signals within the reference region was used to infer the deletion in the *APOBEC3A/B* gene. Homozygous deletion, heterozygous deletion and diploids were identified using the cutoff values of <− 1, − 0.2 and 0, respectively. For samples that predicted a non-carrying deletion, we additionally filtered samples with < 50 probes (corresponding to the number of segmented copy number signals) that overlapped with the deletion regions. We observed that few samples predicted carrying deletion with a relatively high expression level of the *APOBEC3B* gene. As such, we filtered the samples that predicted carrying deletion with expression levels of the *APOBEC3B* gene higher than the median value in samples for each cancer type. Specifically, we removed samples predicted to carry heterozygous deletion in bladder (*N* = 9), breast (*N* = 36), cervical (*N* = 2), lung adenocarcinoma (*N* = 12), lung squamous carcinoma (*N* = 1), head and neck (*N* = 9), stomach (*N* = 3), pancreas (*N* = 1), thyroid (*N* = 8), and kidney (*N* = 5), while no samples predicted to carry homozygous deletion were filtered. Additionally, somatic copy number alterations for breast cancer were downloaded from the cBioPortal (http://www.cbioportal.org/). The somatic copy number alterations for the *APOBEC3A* and *APOBEC3B* genes were extracted from the copy number data. We removed samples identified with somatic copy number alterations in our downstream analysis. Specifically, we removed samples predicted to have somatic *APOBEC3A* or *APOBEC3B* copy number alterations in bladder (*N* = 46), breast (*N* = 86), cervical (*N* = 22), lung adenocarcinoma (*N* = 62), lung squamous carcinoma (*N* = 77), head and neck (*N* = 103), stomach (*N* = 4), pancreas (*N* = 4), thyroid (*N* = 1), and kidney (*N* = 1). In the end, we analyzed a total of 2556 samples that were reliably predicted to carry deletions or to have no deletions in the bladder (*N* = 286), breast (*N* = 734), cervical (*N* = 133), lung adenocarcinoma (*N* = 363), lung squamous carcinoma (*N* = 85), head and neck (*N* = 323), stomach (*N* = 66), pancreas (*N* = 90), thyroid (*N* = 333), and kidney (*N* = 143).

### The analysis of prediction neoantigen loads

Neoantigen load for each sample in TCGA has been characterized by The Cancer Immunome Atlas (TCIA, https://tcia.at/). Charoentong and colleagues characterized the genome-wide Neoantigen landscape for each sample by analyzing RNA-sequencing and whole-exome data from TCGA. In brief, mutational neoantigens were predicted by the use of HLA typing and MHC class I/II binding capabilities. The established neoantigen prediction algorithm NetMHCcons [32] was applied to missense somatic mutations to estimate their binding affinity to the HLA alleles. More detailed analysis processing has been described in previous literature [33]. We downloaded the number of neoantigen loads for each sample from TCIA and applied log2 transfer to fit a better distribution.

### Prediction of the abundance of relative immune cell compositions in TILs

To predict the abundance of immune cell compositions in TIL, we used the normalized expression data for each cancer type from GDAC. We applied Cell Type Identification by Estimating Relative Subsets of known RNA Transcripts (CIBERSORT) to estimate the abundance of each of the 22 immune cell types (i.e. T cells CD4 naive, T cells CD4 memory activated and Tregs) in each tumor tissue sample, based on the normalized expression data of 547 genes [34]. This analysis was implemented in a Stanford University server (https://cibersort.stanford.edu/). Only samples inferred with an abundance of immune cell compositions in TILs at *P* < 0.2 remained for the downstream analysis, as recommended by previous literature [34].

### Pathway enrichment analysis

To identify genes co-expressed with the isoform uc011aoc, we performed a correlation analysis using Rank-based Spearman approach for samples predicted to carry with germline *APOBEC3A/B* deletions for each cancer type. We further performed functional enrichment analysis for the top 100 correlated genes using the Ingenuity Pathway Analysis (IPA) tool (http://www.ingenuity.com/). The top five significant canonical signaling pathways were presented.

### Statistical analysis

We first applied the univariate analyses to evaluate the association of APOBEC-mutational signature with expression levels of APOBEC3A and APOBEC3B genes, and their isoforms. We then include all six isoforms as independent variables in the same models for mutual adjustment for each cancer type. Because the distribution of APOBEC-signature mutation is severely right skewed, the ordinal regression models implemented in the ‘orm’ function from the ‘rms’ library of the R package were used. To elucidate whether germline APOBEC3A/B deletion affects APOBEC-signature mutation, the above models were constructed as following: APOBEC-mutational signature ~ germline APOBEC3A/B deletion; APOBEC-mutational signature ~ germline APOBEC3A/B deletion + uc003awn + uc003awo + uc011aoc (Expression levels); proportion of APOBEC-mutational signature ~ germline APOBEC3A/B deletion; and proportion of APOBEC-mutational signature ~ germline APOBEC3A/B deletion + uc003awn + uc003awo + uc011aoc (Expression levels). To investigate the effects of germline APOBEC3A/B deletion on neoantigen loads and immune cell compositions in TILs, linear regression analyses were conducted by cancer type. Additionally, we used the Wilcoxon signed-rank test to compare the differences of immune cell compositions between the samples with and without germline APOBEC3A/B deletion Finally, the association between APOBEC-signature mutation and neoantigen load was analyzed with univariate linear regression models for each cancer type. All statistical analyses were conducted using the R software.

## Results

### Distinct patterns of the *APOBEC3 genes,* associated with APOBEC-mutational signature in multiple cancer types

Following the previous analysis of APOBEC-mutational signature, we measured the mutations using the number of deaminase C that were within the TCW trinucleotide motif change to T or G mutations per each sample across 10 cancer types [1, 9] (see Methods). We conducted univariate analyses to evaluate associations of APOBEC-mutational signature with the overall gene expression levels of *APOBEC3A* and *APOBEC3B*. We observed that *APOBEC3A* expression level was positively associated with APOBEC-mutational signature in a total of six cancer types – bladder, breast, cervical, lung adenocarcinoma, head and neck, and thyroid. No significant associations were observed in the remaining cancer types, although the same association directions were observed (Additional file 1: Table S1). Interestingly, we observed that *APOBEC3B* expression level was positively associated with APOBEC-mutational signature in all cancer types, except for lung squamous carcinoma (Additional file 1: Table S1). In comparison with the associations from *APOBEC3A*, *APOBEC3B* was specifically associated with APOBEC-mutational signature in stomach, pancreas and kidney cancers, with a *P* = 5.2 × 10^− 11^, *P* = 2.0 × 10^− 3^, and *P* = 1.1 × 10^− 4^, respectively (Additional file 1: Table S1). These findings were in line with previous studies [4, 9, 11, 13]. In addition, we also evaluated the associations of APOBEC-mutational signature with the gene expression *of other APOBEC3* genes: *APOBEC3C, APOBEC3D, APOBEC3F, APOBEC3G,* and *APOBEC3H.* Our results showed that expressions of these genes were associated with APOBEC-mutational signature varied in distinct cancer types (Additional file 1: Table S2). For example, the expression *APOBEC3C* was associated with increased APOBEC-mutational signature in cervical and head and neck cancers, while its expression was associated with decreased APOBEC-mutational signature in stomach cancer. Our result also showed that the expression *APOBEC3D, APOBEC3F, and APOBEC3G* were associated with increased APOBEC-mutational signature, whereas the result from the association of *APOBEC3G* was in line with the previous finding [35]. Interestingly, we observed that expression *APOBEC3H* was associated with increased APOBEC-mutational signature in cervical, but not observed in breast cancer.

### Distinct patterns of the isoforms of *APOBEC3A* and *APOBEC3B,* associated with APOBEC-mutational signature in multiple cancer types

**Fig. 1:**
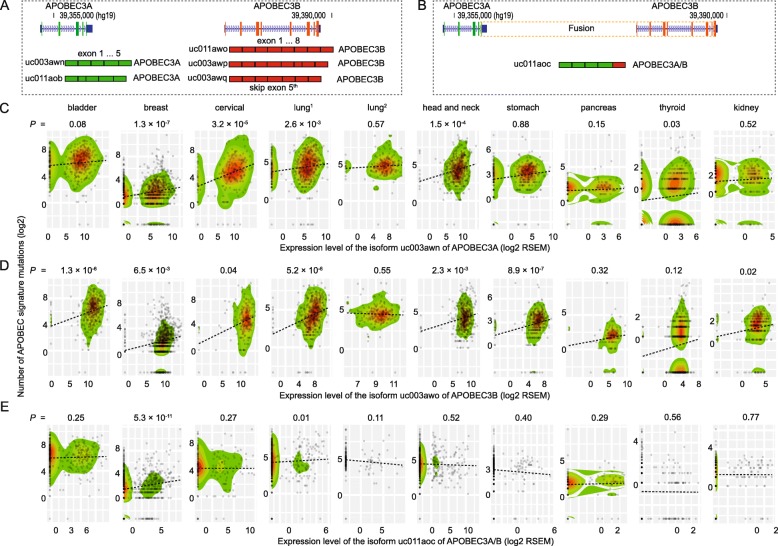
Distinct patterns of the isoforms of *APOBEC3A* and *APOBEC3B* associated with APOBEC-mutational signature. **a** Two isoforms, uc003awn and uc011aob, transcribed from *APOBEC3A*, and three isoforms, uc003awo, uc003awp and uc003awq, transcribed from *APOBEC3B*. **b** Isoform uc011aoc derived from a fusion event covering from the last intronic of *APOBEC3*A to the last exon of *APOBEC3B*. **c**, **d**, **e** Distribution plots indicate the association between APOBEC-mutational signature and isoforms, uc003awn (**c**), uc003awo (**d**) and uc011aoc (**e**)

We further evaluated associations between APOBEC-mutational signature and expression levels of each of the isoforms transcribed from *APOBEC3A* and *APOBEC3B*. A total of six isoforms were analyzed, including uc003awn and uc011aob transcribed from *APOBEC3A*, uc003awo, uc003awp and uc003awq transcribed from *APOBEC3B*, and another isoform, uc011aoc, derived from a fusion event involved in a region covering the last intronic of *APOBEC3A* to the last exon of *APOBEC3B* (Fig. 1a, b). We confirmed that the isoforms uc003awn and uc003awo were primarily transcribed from *APOBEC3A* and *APOBEC3B*, respectively (Additional file 1: Figure S1) [11]. As expected, the association direction of both the uc003awn and uc003awo expression levels and APOBEC-mutational signature were consistent with the observations of overall gene expression levels across all cancer types (Fig. 1c, d; Additional file 1: Table S3). Surprisingly, the expression level of uc011aoc (*APOBEC3A/B*) was positively associated with the APOBEC-mutational signature only in breast cancer (*P* = 5.0 × 10^− 10^) (Fig. 1e; Additional file 1: Table S3). However, no associations for the remaining isoforms were observed in any cancer types, except for a weak association observed for uc011aob (*APOBEC3A*) with head and neck cancer (*P* = 0.02; Additional file 1: Table S3).

Using multiple regression analyses that included all isoforms of both *APOBEC3A* and *APOBEC3B*, we found that the expression levels of uc003awn (*APOBEC3A*) and uc003awo (*APOBEC3B*) were independently and commonly associated with evaluated APOBEC-mutational signature in multiple cancer types: bladder (a marginal association for uc003awn), breast, cervical, lung adenocarcinoma, and head and neck (Table 1). Likewise, an additional association for uc011awn (*APOBEC3A*) was observed in thyroid, while associations for uc003awo (*APOBEC3B*) were observed in stomach, pancreas and kidney cancers. Consistent with the univariate analysis, a striking association for uc011aoc (*APOBEC3A/*B) with APOBEC-mutational signature was observed in breast cancer (*P* = 5.3 × 10^− 11^), and an additional association was also observed in lung adenocarcinoma (*P* = 0.01) (Table 1). These findings suggest that uc011aoc plays a tissue-specific role in affecting APOBEC-mutational signature primarily in breast cancer, while uc003awn and uc003awo play a ubiquitous but distinct role in the entire spectrum of human cancer. Notably, our additional analysis showed the positive expression correlation between the isoform uc011aoc and *APOBEC3A* across a major of cancer types, especially in breast cancer (Additional file 1: Table S4).

**Table 1:** Associations between APOBEC-mutational signature and isoform expression levels of *APOBEC3A* and *APOBEC3B*

Cancer type^a^	uc003awn		uc011aoc		uc011awo	
	Beta	*P*	Beta	*P*	Beta	*P*
bladder	0.035	0.08	0.032	0.25	0.130	1.3 × 10^− 6^
breast	0.085	1.3 × 10^− 7^	0.159	5.3 × 10^− 11^	0.053	6.5 × 10^− 3^
cervical	0.105	3.2 × 10^− 5^	0.035	0.27	0.076	0.04
lung^b^	0.076	2.6 × 10^− 3^	0.097	0.01	0.131	5.2 × 10^− 6^
lung^c^	0.021	0.57	−0.072	0.11	−0.037	0.55
head and neck	0.549	1.5 × 10^− 4^	−0.013	0.52	0.086	2.3 × 10^− 3^
stomach	−0.004	0.88	−0.050	0.40	0.144	8.9 × 10^−7^
pancreas	0.086	0.15	0.099	0.29	0.076	0.32
thyroid	0.070	0.03	0.043	0.56	0.059	0.12
kidney	0.024	0.52	−0.026	0.77	0.095	0.02

“^a^” Sample size for each cancer type: bladder (*N* = 388), breast (*N* = 961), cervical (*N* = 185), lung adenocarcinoma (*N* = 475), lung squamous cell carcinoma (*N* = 178), head and neck (*N* = 498), stomach (*N* = 368), pancreas (*N* = 119), thyroid (*N* = 485) and kidney (*N* = 280). “^b^” and “^c^” refers to lung adenocarcinoma and lung squamous carcinoma, respectively. A multivariate regression analysis was constructed to include all six isoforms as independent variables and APOBEC-signature mutation as the dependent variable for each cancer type. Multivariate regression analysis was constructed to include all six isoforms as independent variables and APOBEC-signature mutation as the dependent variable for each cancer type. The significance level at *P* = 0.005, corresponding to a threshold with a Bonferroni-correction of *P* = 0.05, given 10 tests

In addition, we performed an association analysis stratified by clinical subtypes in breast cancer. We observed that the associations of the isoforms of uc003awn (APOBEC3A) and uc011aoc (APOBEC3A/B) with APOBEC-mutational signature varied across different clinical subtypes, with the most significant association being observed in the LumA subtype (Additional file 1: Table S5).

### Germline *APOBEC3A/B* deletion affecting expression levels of isoforms of the *APOBEC3A* and *APOBEC3B* genes

**Fig. 2:**
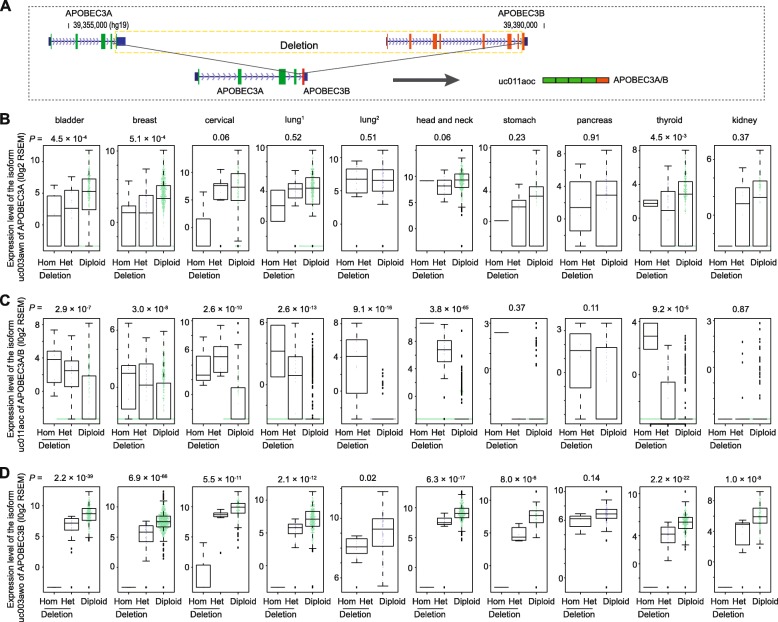
Associations between the isoform expression levels of *APOBEC3A* and *APOBEC3B* and germline *APOBEC3A/B* deletion. **a** Isoform uc011aoc transcribed from a fusion region derived from a germline deletion covering from the last intronic of *APOBEC3A* to the last exon of *APOBEC3B*. **b**, **c**, **d** Box plots indicate expression levels of isoforms, uc003awn (**b**), uc003awo (**c**) and uc011aoc (**d**), for samples that are predicted to carry homozygous and heterozygous deletion and have no deletion for each cancer type

To investigate how germline *APOBEC3A/B* deletion affects expression of the isoforms of *APOBEC3A* and *APOBEC3B*, we first identified 30 samples predicted to carry homozygous deletion, 239 samples predicted to carry heterozygous deletion and 2287 samples predicted to have no deletion (Additional file 1: Table S6, see Methods). Next, we evaluated associations between germline *APOBEC3A/B* deletion and the expression levels of each isoform using univariate analysis (see Methods). As expected, we observed that germline *APOBEC3A/B* deletion was significantly associated with decreased expression levels of the isoform uc003awo (*APOBEC3B)* across all cancer types at *P* < 0.05, except for pancreas cancer with a *P* = 0.14. Significant associations with decreased expression levels of uc003awn (*APOBEC3A)* were also observed in three cancer types – bladder, breast and thyroid (Fig. 2; Table 2). In contrast, our results showed that germline *APOBEC3A/B* deletion was significantly associated with an increased expression level of uc011aoc (*APOBEC3A/B*) across all cancer types, except for stomach, pancreas and kidney cancers. For these, there was no statistical significance but had the same association directions (Fig. 2; Table 2). In particular, head and neck cancer showed the most significant association with *P* = 3.8 × 10^− 65^, and breast and bladder cancers showed the significant association with *P* = 3.0 × 10^− 8^, and *P* = 2.9 × 10^− 7^, respectively. Additionally, we performed the same analysis, stratified by population, and a similar trend was observed in these cancer types (data not shown). Our findings suggest that, in almost all investigated cancer types, germline *APOBEC3A/B* deletion was significantly associated with decreased expression levels of uc003awn and uc003awo, but there was an increased expression level of uc011aoc.

**Table 2:** Associations between isoform expression levels of *APOBEC3A* and *APOBEC3*B and germline *APOBEC3A/B* deletion

Cancer type^a^	uc003awn		uc011aoc		uc011awo	
	Beta	*P*	Beta	*P*	Beta	*P*
bladder	1.52	4.5 × 10^− 4^	−1.31	2.9 × 10^−7^	3.42	2.2 × 10^− 39^
breast	0.92	5.1 × 10^− 4^	− 0.71	3.0 × 10^− 8^	3.03	6.9 × 10^− 66^
cervical	1.44	0.06	−2.79	2.6 × 10^− 10^	2.67	5.5 × 10^− 11^
lung^b^	0.28	0.52	−1.35	2.6 × 10^− 13^	2.33	2.1 × 10^− 12^
lung^c^	0.55	0.51	− 3.60	9.1 × 10^− 16^	1.08	0.02
head and neck	0.86	0.06	−5.74	3.8 × 10^− 65^	2.63	6.3 × 10^− 17^
stomach	1.05	0.23	−0.25	0.37	3.01	8.0 × 10^− 6^
pancreas	−0.14	0.91	−1.00	0.11	0.95	0.14
thyroid	0.90	4.5 × 10^−3^	−0.38	9.2 × 10^−5^	2.13	2.2 × 10^−22^
kidney	0.43	0.37	0.02	0.87	2.26	1.0 × 10^−8^

“^a^” Sample size for each cancer type: bladder (*N* = 286), breast (*N* = 734), cervical (*N* = 133), lung adenocarcinoma (*N* = 363), lung squamous carcinoma (*N* = 85), head and neck (*N* = 323), stomach (*N* = 66), pancreas (*N* = 90), thyroid (*N* = 333), kidney (*N* = 143). “^b^” and “^c^” refers to lung adenocarcinoma and lung squamous carcinoma, respectively. A univariate regression analysis was constructed to include APOBEC-mutational signature as dependent variables and expression levels as the independent variable for each isoform of each cancer type. The significance level at *P* = 0.005, corresponding to a threshold with a Bonferroni-correction of *P* = 0.05, given 10 tests

### Germline *APOBEC3A/B* deletion influencing APOBEC-mutational signature, neoantigen loads and relative immune cell compositions, specifically in breast cancer

**Fig. 3:**
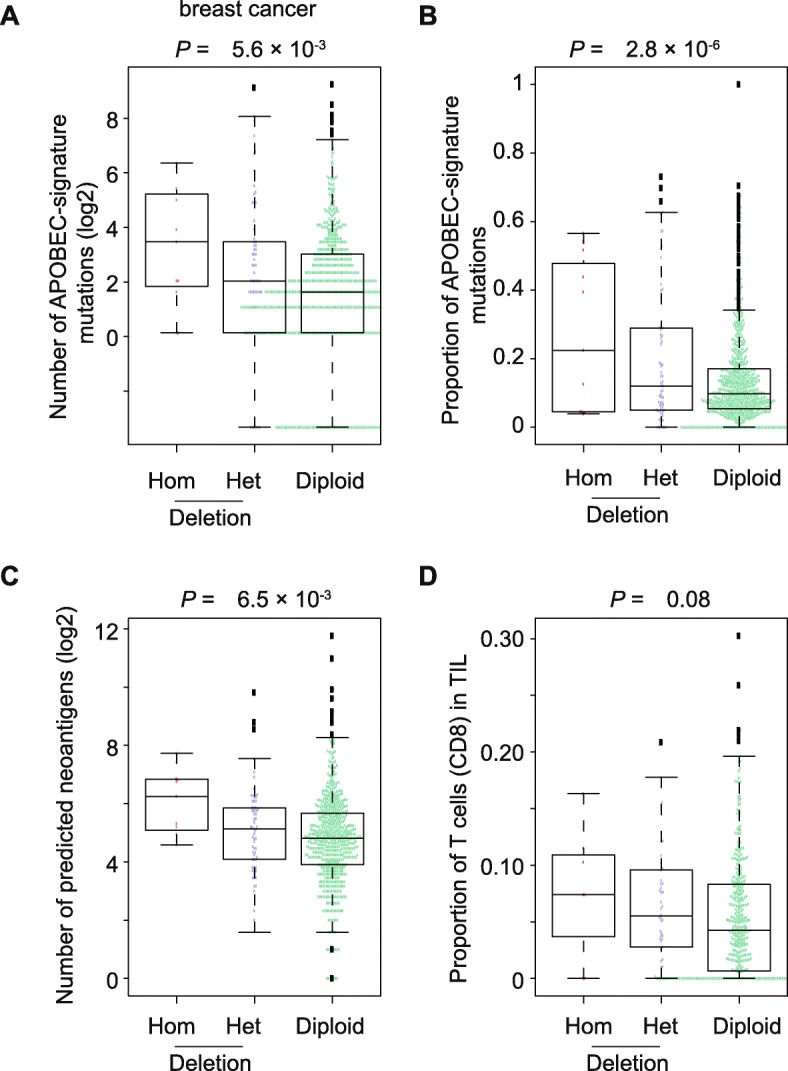
Germline *APOBEC3A/B* deletion associated with APOBEC-mutational signature, neoantigen loads and relative composition of T cells (CD8+) in breast cancer. A significantly higher absolute (**a**) and relative (**b**) APOBEC-mutational signature, neoantigen loads (**c**) and relative composition of T cells (CD8+) (**d**) in samples predicted to carry homozygous and heterozygous deletion compared to samples predicted to have no deletion in breast cancer

A previous study showed that germline *APOBEC3A/B* deletion is associated with increased APOBEC-mutational signature in breast cancer, while a similar pattern, but without statistical significance, was observed in many other cancer types, such as bladder [3]. Using univariate analysis to evaluate the overall effects of germline *APOBEC3A/B* deletion on APOBEC-mutational signature, we found that the deletion was significantly associated with increased APOBEC-mutational signature only in breast cancer (*P* = 5.6 × 10^− 3^; Table 3; Fig. 3a). However, an opposite trend was observed in most other cancer types, although most associations were not statistically significant (Table 3). Specifically, in bladder cancer, we observed that germline *APOBEC3A/B* deletion was significantly associated with decreased APOBEC-mutational signature (*P* = 1.7 × 10^− 3^; Table 3). To further elucidate whether the influence of germline *APOBEC3A/B* deletion on APOBEC-mutational signature is due to its effect on gene expression, we further evaluated associations between APOBEC-mutational signature and the deletion with an adjustment for the expression of isoforms (see Methods). Similarly, our results revealed that germline *APOBEC3A/B* deletion was significantly associated with increased APOBEC-mutational signature only in breast cancer (*P* = 2.8 × 10^− 6^; Table 3; Fig. 3b). A higher effect size (Beta = − 0.620) of the deletion with an adjusted isoform expression was observed when compared to the initial observation of the overall effect (Beta = − 0.281; Table 3). To evaluate whether the germline deletion may contribute to the proportion of APOBEC-mutational signature, we also analyzed a proportion of APOBEC-mutational signature relative to total mutations for each sample (see Methods). Consistent with the initial observation of APOBEC-mutational signature, we observed that the germline deletion was significantly associated with increased proportion of APOBEC-mutational signature only in breast cancer (Beta = − 0.287, *P* = 4.8 × 10^− 3^ and Beta = − 0.5, *P* = 1.4 × 10^− 4^ for the overall effect and effects with adjusted gene expression; see Table 3). These results indicate that germline *APOBEC3A/B* deletion, which leads to increased APOBEC-mutational signature, is likely due to it’s the distinct function of uc011aoc transcribed from the deletion, apart from its effect on increased expressions of uc011aoc. It has been reported that *APOBEC3H* haplotype I (*APOBEC3H-I*) may majorly contribute to APOBEC-mutational signature for samples carrying germline *APOBEC3A/B* deletions in breast cancer [36]. We further analyzed the *APOBEC3H-I* haplotype for a total of 76 samples that were predicted to carry germline *APOBEC3A/B* deletions (see Methods). Our results showed that APOBEC-mutational signature was not significantly correlated with the *APOBEC3H-I* haplotype, regardless of the samples predicted to carry homozygous or heterozygous germline *APOBEC3A/B* deletions (Data not shown). However, our findings are in line with the previous finding that the APOBEC3A/B protein, generated by the deletion, has a higher expression level than the APOBEC3A protein based on the investigation from in vitro functional assays [2].

**Table 3:** Associations between APOBEC-mutational signature and germline *APOBEC3A/B* deletion

Cancer type^a^	S ~ D^d^		S ~ D + E^e^		PS ~ D^f^		PS ~ D + E^g^	
	Beta	*P*	Beta	*P*	Beta	*P*	Beta	*P*
bladder	0.430	1.7 × 10^− 3^	0.082	0.68	0.354	9.4 × 10^− 3^	0.000	0.99
breast	−0.281	5.6 × 10^− 3^	−0.620	2.8 × 10^− 6^	− 0.287	4.8 × 10^− 3^	− 0.50	1.4 × 10^− 4^
cervical	0.281	0.19	0.017	0.96	0.278	0.20	−0.063	0.84
lung^b^	−0.048	0.78	−0.282	0.07	−0.124	0.48	−0.449	0.03
lung^c^	0.511	0.13	0.077	0.88	0.385	0.25	−0.190	0.70
head and neck	0.160	0.42	−0.067	0.85	0.284	0.16	0.065	0.85
stomach	−0.141	0.67	−0.319	0.42	−0.060	0.86	−0.074	0.85
pancreas	−0.439	0.39	−0.571	0.29	−0.388	0.45	−0.498	0.36
thyroid	0.029	0.85	−0.238	0.19	0.025	0.87	−0.231	0.20
kidney	0.323	0.17	0.14	0.59	0.292	0.21	0.105	0.69

“^a^” Sample size for each cancer type: bladder (N = 286), breast (N = 734), cervical (N = 133), lung adenocarcinoma (N = 363), lung squamous carcinoma (N = 85), head and neck (N = 323), stomach (N = 66), pancreas (N = 90), thyroid (N = 333), kidney (N = 143). “^b^” and “^c^” refers to lung adenocarcinoma and lung squamous carcinoma, respectively. “^d^”: APOBEC-mutational signature ~ Germline *APOBEC3A/B* deletion; “^e^”: APOBEC-mutational signature ~ Germline *APOBEC3A/B* deletion + uc003awn + uc003awo + uc011aoc (Expression levels); “^f^”: Proportion of APOBEC-mutational signature ~ Germline *APOBEC3A/B* deletion; “^g^”: Proportion of APOBEC-mutational signature ~ Germline *APOBEC3A/B* deletion + uc003awn + uc003awo + uc011aoc (Expression levels); In association models: “S” refers to APOBEC-mutational signature; “D” refers to germline APOBEC3A/B deletion; “E” refers to isoform expression levels; “RS” refers to proportion APOBEC-mutational signature. The significance level at *P* = 0.005, corresponding to a threshold with a Bonferroni-correction of *P* = 0.05, given 10 tests

To further explore the distinct function and potential pathways that the isoform uc011aoc expression (transcribed from the *APOBEC3A/B* deletion) may be involved in, we analyzed genes that were co-expressed with the isoform uc011aoc in the samples predicted to carry germline APOBEC3A/B deletions (see Methods). An enrichment analysis in canonical signaling pathways using IPA revealed that these co-expressed genes were significantly enriched in distinct canonical pathways varied across cancer types. Specifically, we observed that the top enriched pathways were HIPPO signaling for bladder, PTEN signaling for breast, iNOS and Interferon signaling for lung adenocarcinoma, Acyl-CoA Hydrolysis for stomach, and DNA Double-Strand Break Repair and Fatty Acid α-oxidation for pancreas, EIF2 signaling for thyroid and GPCR-Mediated Integration for kidney (*P* < 0.01 for all; Additional file 1: Table S7). Additionally, a functional enrichment in Cell Death and Survival was commonly observed in multiple cancer types including bladder, breast, cervical, lung adenocarcinoma, and lung carcinoma (*P* < 0.05 for all).

We further evaluated the association between germline *APOBEC3A/B* deletion and neoantigen loads. Consistent with the observation of APOBEC-mutational signature, our results showed that germline *APOBEC3A/B* deletion was significantly associated with increased neoantigen loads only in breast cancer (*P* = 6.5 × 10^− 3^; Fig. 3c), while an opposite trend was observed in many other cancer types (Additional file 1: Table S8). Similarly, we found that the germline deletion was marginally associated with the relative abundance of the composition of T cells (CD8+) in TILs, but only in breast cancer (*P* = 0.08; Fig. 3d). The significant association was detected when we combed samples with both homozygous and heterozygous deletions and compared them to the samples with non-carrying deletions (a Wilcoxon signed-rank test, *P* < 0.05). However, no association was observed for other immune cells. Our findings showed that germline *APOBEC3A/B* deletion plays a tissue-specific role in affecting APOBEC-mutational signature and immunogenicity in breast cancer, likely reinforcing the findings in previous genome wide association studies of potential mechanisms for their association with increased breast cancer risk.

### APOBEC-mutational signature significantly contributing to neoantigens

**Fig. 4:**
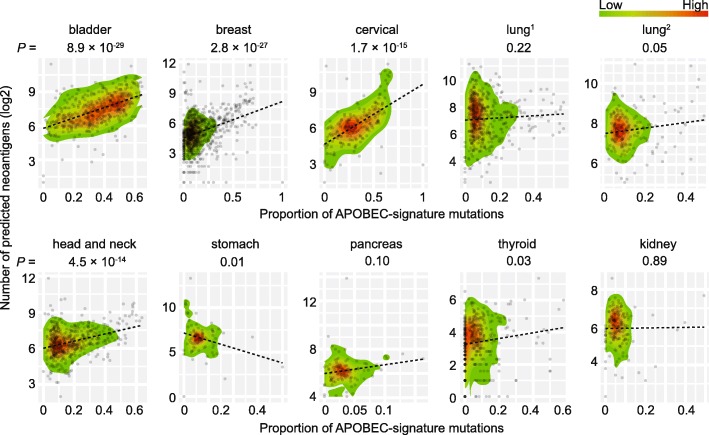
Associations between predicted neoantigen loads and proportion of APOBEC-mutational signature

To investigate to what extent APOBEC-mutational signature contribute to neoantigens, we analyzed predicted neoantigen loads for each sample collected from a previous study [33] (see Methods). Using univariate analysis, we evaluated associations between APOBEC-mutational signature and neoantigen loads for each cancer type. As expected, APOBEC-mutational signature was positively associated with neoantigen loads in all cancer types (*P* < 1.0 × 10^− 4^ for all comparisons), whereas the top significant associations were observed in breast and bladder types with *P* = 5.1 × 10^− 125^, and *P* = 1.5 × 10^− 90^, respectively (Additional file 1: Table S9). Similarly, an overall positive association trend was observed between predicted neoantigen loads and proportion of APOBEC-mutational signature, with the exception of stomach cancer (Fig. 4; Additional file 1: Table S10). Specifically, the significant associations were observed in multiple cancer types, including bladder, breast, cervical, lung adenocarcinoma, head and neck, and thyroid. In particular, breast and bladder cancer showed the best associations with *P* = 8.9 × 10^− 29^, and *P* = 2.8 × 10^− 27^, respectively (Additional file 1: Table S10). Our findings suggest that APOBEC-mutational signature play a significant role in contributing to the biogenesis of neoantigens in human cancer.

### Associations between relative abundance of immune cell compositions in TILs with neoantigen load and APOBEC-mutational signature

To investigate the relationship between neoantigen load and TILs, we used gene expression data in tumor tissues to measure the abundance of relative cell compositions of each immune cell type, including B cell naïve, B cell memory, T cell CD8 and T cells CD4 memory-activated in TILs (see Methods). Using univariate analysis, we evaluated the association between neoantigen load and the relative abundance of immune cell compositions for each cancer type. We observed that there was a positive association trend between neoantigen loads and both T cell CD8+ and CD4+ memory-activated types in all cancer types except thyroid and kidney (Binomial test *P* = 0.11 and *P* = 0.02 for T cell CD8+ and CD4+ memory-activated types, respectively). An opposite pattern was observed for both B cell naïve and memory types across all cancer types, with the exception of lung adenocarcinoma (Binomial test *P* = 0.02 and *P* = 2.2 × 10^− 16^ for B cell naïve and memory types, respectively). In particular, our results showed that neoantigen loads had an association with the relative abundances of both T cell CD8+ and CD4+ memory-activated, and B cell naïve and memory types in bladder cancer. There was an association for both B cell naïve and memory types in breast cancer, and T cell CD4 memory- activated cell types in lung adenocarcinoma, head and neck, and pancreas cancers (Additional file 1: Table S11). As APOBEC-mutational signature significantly contributed to neoantigens, we additionally evaluated the association between the relative abundances of the immune cell compositions in TILs and APOBEC-mutational signature. Consistent with the observation from association analysis of naeoantigen load, a similar pattern was also found for APOBEC-mutational signature (Additional file 1: Table S12). Our findings suggest that APOBEC-mutational signature have an influence on cancer immunogenic abilities, such as attracting certain immune cells in TILs, possibly mediated by the increased neoantigen load.

## Discussion

In this pan-cancer study, we systematically analyzed APOBEC-mutational signature in relation to isoform expression and germline *APOBEC3/B* deletion. Our study showed that the isoforms uc003awn (*APOBEC3A*) and uc003awo (*APOBEC3B*) were independently associated with a higher burden of APOBEC-mutational signature in multiple cancer types, while such an association for the uc011aoc (*APOBEC3A/B*) was only observed in breast cancer. We also found that, across cancer types, the germline *APOBEC3A/B* deletion led to decreased expression levels of uc003awn and uc003awo but caused an increased expression level of uc011aoc. Furthermore, our results indicate that germline *APOBEC3A/B* deletion leading to increased APOBEC-mutational signature is likely due to a distinct function of the isoform uc011aoc transcribed from the *APOBEC3A/B* chimera, apart from its effect on an increased expression of uc011aoc. Our findings provide novel insight into understanding the APOBEC biological mechanisms involved in carcinogenesis.

The investigation of the relationship between germline *APOBEC3A/B* deletion and APOBEC-mutational signature has been well-studied. For example, Nik-Zainal and colleagues showed that the deletion was associated with increased APOBEC-mutational signature in breast cancer. They also concluded that this pattern may exist ubiquitously in other cancer types (e.g., bladder cancer) [3]. Their findings significantly contributed to the understanding of the association of germline deletion with breast cancer risk [20, 23]. Recently, Middlebrooks and colleagues analyzed the expression level of the APOBEC3A/B deletion isoform as a proxy to evaluate the association between germline *APOBEC3A/B* deletion and APOBEC-mutational signature in breast and bladder cancer. They suggested that the expression of the deletion isoform (uc011aoc) was associated with evaluated APOBEC-mutational signature in breast cancer, but not in bladder cancer [11]. In our study, we refined the analysis to identify samples predicted to carry deletions, and samples predicted to have no deletions, by integrating the deletion data from the sequencing data of a previous study [3] with an analysis of array-based genotype and gene expression data (see Methods). In comparison to previous analyses, we used a strategy to strictly control data quality and to filter samples with ambiguous deletion calling by introducing additional multiple datasets. In particular, we filtered a few samples that were predicted to carry a deletion with a relatively high expression level of the *APOBEC3B* gene (uc003awo). On the other hand, we performed multiple regression analyses that included all isoforms in order to evaluate uc011aoc (*APOBEC3A/B*) with APOBEC-mutational signature, to address the analysis challenge of introducing potential confounders due to the complexity of alternative splicing. Our results indicate that the expression level of uc011aoc derived from germline *APOBEC3A/B* deletion plays a tissue-specific functional influence on APOBEC-mutational signature in breast cancer. Notably, the expression level of the isoform uc011aoc in tumor tissues may not fully reflect the level in premalignant tissues due to possible factors, such as tumor heterogeneity and potential confounders. In addition, the expression level of the isoform uc011aoc was measured based on RNA-seq data. Future experiment using quantitative PCR (qPCR) is needed to further verify the expression level of isoform uc011aoc from RNA-seq data. Nevertheless, our results showed a strong association between the expression of the isoform and germline *APOBEC3A/B* deletion in breast and other cancer types, indicating the reliability of these findings. Consistent with this observation, our results further showed that germline *APOBEC3A/B* deletion led to increased APOBEC-mutational signature in breast cancer, providing an explanation that germline *APOBEC3A/B* deletion is associated with increased breast cancer risk. These findings provide new insight into the understanding of the deletion that is associated with increased breast cancer risk.

In the analysis of associations between isoforms and mutational signature, we only focused on APOBEC-signature mutation (TCW - > T/G). The background signatures should not affect our analysis, as they are in distinct mutation patterns (i.e. smoking-related mutational signature with A - > C) with APOBEC-mutational signature [6, 37, 38]. Although the statistical power varied across cancer types due to different sample sizes and different proportions of germline APOBEC3A/B deletion carriers, our result showed that the association of uc011aoc (APOBEC3A/B) with APOBEC-mutational signature observed in breast cancer was stood out, which was 1.6 ~ 5 times of the associations compared to other cancer types (Table 1).

Immunotherapies, such as the suppression of immune checkpoints (αPD-1, αPD-L1), have revolutionized the treatment of human cancers [39–45]. The immune checkpoints (i.e. PD-1, PD-L1 and CTLA-4) and other immune-related and mismatch repair (MMR) genes, together with TILs and neoantigens, play critical roles in anti-cancer immunoreactivity. They have been linked to immunotherapy outcomes. In particular, previous studies have shown that an overall mutation load is highly correlated with neoantigens and TILs [43, 45, 46]. A recent study showed that APOBEC-mutational signature was associated with the increasing of neo-peptide hydrophobicity [47]. In line with these findings, our findings suggest that APOBEC-mutational signature, in relation to expression and germline deletion of *APOBEC* genes, substantially contribute to neoantigens and, consequently, affect certain immune cell compositions in TILs. In addition, recent studies suggest that *APOBEC* plays an important role in promoting PD-1, as well as immune activation in multiple cancer types, implying its potential for cancer immunotherapy [25, 29, 30, 48]. Thus, our findings, together with other studies, highlight the importance of the *APOBEC* genes in immunogenicity and cancer immunotherapy.

## Conclusions

In conclusion, our results showed that uc011aoc, primarily generated from germline *APOBEC3A/B* deletion, plays a tissue-specific role in promoting APOBEC-mutational signature. We further showed that germline *APOBEC3A/B* deletion influences APOBEC-mutational signature, neoantigen loads and the relative abundance of T cell (CD8+) composition, but only in breast cancer. These functional consequences of the germline deletion are likely due to a distinct function of the isoform uc011aoc transcribed from the *APOBEC3A/B* chimera, apart from its induced expression level. These findings provide potential mechanisms for understanding the association of germline *APOBEC3A/B* gene deletion with cancer risk. Our results also showed that APOBEC-mutational signature significantly contribute to neoantigens, and consequently attract certain immune cells in TILs ubiquitously observed in human cancer. This study provides novel insights into understanding the genetic, biological and immunological mechanisms through which APOBEC genes may be involved in carcinogenesis.

## Supplementary information

**Additional file 1: Table S1**. Associations between APOBEC-mutational signature and gene expression levels of *APOBEC3A* and *APOBEC3B*. **Table S2**. Associations between APOBEC-mutational signature and gene expression levels of *APOBEC3C, APOBEC3D, APOBEC3F, APOBEC3G,* and *APOBEC3H*. **Table S3**. Associations between APOBEC-mutational signature and each isoform expression level of *APOBEC3A* and *APOBEC3B*. **Table S4**. Expression correlation between *APOBEC3A* with the isoform uc011aoc for each cancer types. **Table S5**. Associations between APOBEC-mutational signature and isoform of *APOBEC3A* and *APOBEC3B* stratified by clinical subtypes in breast cancer. **Table S6**. The distribution of deletion genotypes in samples for each cancer type. **Table S7**. A list of top enriched canonical pathways for genes that were co-expressed with the isoform uc011aoc across cancer types. **Table S8**. Associations between predicted neoantigen loads and germline *APOBEC3A/B* deletion. **Table S9**. Associations between predicted neoantigen loads and APOBEC-mutational signature. **Table S10**. Associations between predicted neoantigen loads and proportion of APOBEC-mutational signature. **Table S11.** Associations between abundance of relative immune cell compositions in TILs and neoantigen loads. **Table S12**. Associations between abundance of relative immune cell compositions in TILs and APOBEC-mutational signature. **Figure S1**. The expression levels of six isoforms of *APOBEC3A* and *ABOBEC3B* for each cancer type.

## Data Availability

Main source R codes that are used in this work are available from Github (https://github.com/XingyiGuo/APOBEC/tree-save/master/Associations/APOBEC-Sig). Completed data sets, which included expressions of genes (RNAseqv2, Level_3, RSEM_genes_normalized) and isoforms (RNAseqv2, Level_3, RSEM_isoforms_ normalized), APOBEC mutational signatures (Mutation_APOBEC, Level_4) and the segmented copy number variations (Merged, genome_wide_snp_6, Level_3, segmentation, hg19) were downloaded from the TCGA using the Broad Institute Genome Data Analysis Center (GDAC) Firehose portal through Firebrowse (stamp data/analyses__2016_01_28, http://gdac.broadinstitute.org). The human gene annotation data set was obtained from the Table Browser of the UCSC genome browser (https://genome.ucsc.edu/cgi-bin/hgTables). The six isoforms that were investigated include uc003awn, uc011aob, uc011aoc, uc003awo, uc003awp and uc003awq, transcribed from the genes APOBEC3A and APOBEC3B.

## Abbreviations

APOBEC: Apolipoprotein B mRNA editing enzyme, catalytic polypeptide-like
BCAC: The Breast Cancer Association Consortium (BCAC)
CIBERSORT: Estimating Relative Subsets of known RNA Transcripts
CTLs: Cytotoxic T-cells
GWAS: Genome-wide association studies
IPA: Ingenuity Pathway Analysis (IPA)
MHC: Major histocompatibility complex (MHC)
MMR: Mismatch repair
RSEM: RNA-Seq by Expectation Maximization
SNP: Single nucleotide polymorphisms (SNPs)
TCGA: The Cancer Genome Atlas
TCIA: The Cancer Immunome Atlas
TILs: Tumor infiltration lymphocytes
