# Supplementary material for: Integrative genomic analyses of APOBEC-mutational signature, expression and germline deletion of APOBEC3 genes, and immunogenicity in multiple cancer types
Source: BMC Med Genomics. 2019 Sep 18;12:131. doi: 10.1186/s12920-019-0579-3 (PMC6751822; doi:10.1186/s12920-019-0579-3)

**Table S1:** Associations between APOBEC-mutational signature and gene expression levels of *APOBEC3A* and *APOBEC3B*.

| **Cancer type^*^** | ***APOBEC3A*** | | ***APOBEC3B*** | |
| --- | --- | --- | --- | --- |
|  | **Beta** | ***P*** | **Beta** | ***P*** |
| bladder | 0.098 | 6.4 × 10^-5^ | 0.125 | 8.4 × 10^-9^ |
| breast | 0.134 | 2.2 × 10^-16^ | 0.097 | 3.9 × 10^-9^ |
| cervical | 0.177 | 8.5 × 10^-9^ | 0.135 | 4.2 × 10^-4^ |
| lung^1^ | 0.181 | 1.0 × 10^-11^ | 0.171 | 2.1 × 10^-12^ |
| lung^2^ | 0.045 | 0.26 | -0.003 | 0.95 |
| head and neck | 0.126 | 1.1 × 10^-8^ | 0.138 | 1.7 × 10^-5^ |
| stomach | 0.036 | 0.07 | 0.209 | 5.2 × 10^-11^ |
| pancreas | 0.028 | 0.53 | 0.231 | 2.0 × 10^-3^ |
| thyroid | 0.081 | 7.6 × 10^-4^ | 0.068 | 0.01 |
| kidney | 0.031 | 0.25 | 0.178 | 1.1 × 10^-4^ |

“*” Sample size for each cancer type: bladder (N = 286), breast (N = 734), cervical (N = 133), lung adenocarcinoma (N = 363), lung squamous carcinoma (N = 85), head and neck (N = 323), stomach (N = 66), pancreas (N = 90), thyroid (N = 333), kidney (N = 143). “^1^” and “^2^” refers to lung adenocarcinoma and lung squamous carcinoma, respectively. The significance level at *P* = 0.005, corresponding to a threshold with a Bonferroni-correction of *P* = 0.05, given 10 tests.

**Table S2:** Associations between APOBEC-mutational signature and gene expression levels of *APOBEC3C, APOBEC3D, APOBEC3F, APOBEC3G,* and *APOBEC3H*.

| **Cancer type^*^** | ***APOBEC3C*** | | ***APOBEC3D*** | | ***APOBEC3F*** | | ***APOBEC3G*** | | ***APOBEC3H*** | |
| --- | --- | --- | --- | --- | --- | --- | --- | --- | --- | --- |
|  | **Beta** | ***P*** | **Beta** | ***P*** | **Beta** | ***P*** | **Beta** | ***P*** | **Beta** | ***P*** |
| bladder | 0.004 | 0.93 | 0.014 | 0.74 | 0.121 | 0.02 | 0.075 | 0.04 | 0.036 | 0.08 |
| breast | 0.054 | 0.11 | 0.091 | 2.3 × 10^-3^ | 0.113 | 1.5 × 10^-3^ | 0.110 | 1.6 × 10^-4^ | 0.021 | 0.12 |
| cervical | 0.292 | 2.1 × 10^-4^ | 0.135 | 0.02 | 0.044 | 0.61 | 0.155 | 0.01 | 0.201 | 2.4 × 10^-4^ |
| lung^1^ | 0.021 | 0.68 | 0.135 | 5.2 × 10^-3^ | 0.134 | 0.01 | 0.069 | 0.1 | 0.021 | 0.3 |
| lung^2^ | -0.104 | 0.3 | 0.044 | 0.51 | -0.044 | 0.62 | 0.044 | 0.53 | 0.028 | 0.64 |
| head and neck | 0.230 | 2.7 × 10^-5^ | 0.115 | 1.2 × 10^-4^ | 0.145 | 7.1 × 10^-4^ | 0.077 | 0.03 | 0.046 | 0.02 |
| stomach | -0.152 | 4.0 × 10^-3^ | -0.016 | 0.74 | -0.049 | 0.38 | -0.035 | 0.41 | -0.004 | 0.84 |
| pancreas | 0.083 | 0.45 | 0.153 | 0.19 | -0.170 | 0.3 | -0.025 | 0.83 | -0.008 | 0.82 |
| thyroid | 0.186 | 0.01 | 0.046 | 0.34 | 0.021 | 0.81 | 0.016 | 0.77 | 0.014 | 0.66 |
| kidney | 0.115 | 0.04 | 0.118 | 0.06 | 0.178 | 0.09 | 0.064 | 0.33 | -0.018 | 0.64 |

“*” Sample size for each cancer type: bladder (N = 286), breast (N = 734), cervical (N = 133), lung adenocarcinoma (N = 363), lung squamous carcinoma (N = 85), head and neck (N = 323), stomach (N = 66), pancreas (N = 90), thyroid (N = 333), kidney (N = 143). “^1^” and “^2^” refers to lung adenocarcinoma and lung squamous carcinoma, respectively. The significant level at *P* = 0.005, corresponding to a threshold with Bonferroni-correction *P* =0.05 given 10 tests. The significance level at *P* = 0.005, corresponding to a threshold with a Bonferroni-correction of *P* = 0.05, given 10 tests.

**Table S3:** Associations between APOBEC-mutational signature and each isoform expression level of *APOBEC3A* and *APOBEC3B*.

| **Cancer type^*^** | **uc003awn** | | **uc011aob** | | **uc011aoc** | | **uc011awo** | | **uc011awp** | | **uc011awq** | |
| --- | --- | --- | --- | --- | --- | --- | --- | --- | --- | --- | --- | --- |
|  | **Beta** | ***P*** | **Beta** | ***P*** | **Beta** | ***P*** | **Beta** | ***P*** | **Beta** | ***P*** | **Beta** | ***P*** |
| bladder | 0.044 | 4.6 × 10^-3^ | -0.001 | 0.96 | 0.021 | 0.40 | 0.136 | 9.9 × 10^-9^ | -0.102 | 0.10 | -0.009 | 0.80 |
| breast | 0.086 | 4.4 × 10^-12^ | -0.034 | 0.08 | 0.145 | 5.0 × 10^-10^ | 0.100 | 9.3 × 10^-10^ | -0.045 | 0.14 | 0.038 | 0.18 |
| cervical | 0.115 | 5.8 × 10^-8^ | -0.030 | 0.36 | 0.010 | 0.74 | 0.115 | 3.6 × 10^-4^ | -0.035 | 0.67 | 0.034 | 0.35 |
| lung^1^ | 0.088 | 2.6 × 10^-6^ | 0.001 | 0.98 | 0.080 | 0.03 | 0.158 | 1.3 × 10^-11^ | -0.073 | 0.08 | -0.047 | 0.16 |
| lung^2^ | 0.045 | 0.13 | -0.083 | 0.05 | -0.085 | 0.05 | -0.005 | 0.93 | -0.048 | 0.76 | 0.089 | 0.08 |
| head and neck | 0.096 | 4.6 × 10^-7^ | 0.043 | 0.02 | -0.019 | 0.29 | 0.105 | 4.6 × 10^-5^ | -0.002 | 0.97 | 0.025 | 0.27 |
| stomach | 0.057 | 6.2 × 10^-3^ | -0.055 | 0.10 | -0.054 | 0.33 | 0.127 | 6.5 × 10^-7^ | 0.085 | 0.14 | -0.070 | 0.22 |
| pancreas | 0.033 | 0.39 | 0.026 | 0.63 | 0.074 | 0.33 | 0.104 | 0.08 | 0.001 | 0.99 | 0.029 | 0.65 |
| thyroid | 0.062 | 5.5 × 10^-3^ | 0.015 | 0.65 | -0.020 | 0.78 | 0.098 | 1.3 × 10^-3^ | -0.039 | 0.43 | -0.026 | 0.59 |
| kidney | 0.034 | 0.25 | 0.011 | 0.78 | 0.005 | 0.95 | 0.097 | 3.7 × 10^-3^ | -0.113 | 0.02 | 0.040 | 0.44 |

“*” Sample size for each cancer type: bladder (N = 286), breast (N = 734), cervical (N = 133), lung adenocarcinoma (N = 363), lung squamous carcinoma (N = 85), head and neck (N = 323), stomach (N = 66), pancreas (N = 90), thyroid (N = 333), kidney (N = 143). “^1^” and “^2^” refers to lung adenocarcinoma and lung squamous carcinoma, respectively. The significant level at *P* = 0.005, corresponding to a threshold with Bonferroni-correction *P* =0.05 given 10 tests.

**Table S4:** Expression correlation between *APOBEC3A* with the isoform uc011aoc for each cancer types.

| **Cancer type*** | **Correlation coefficient** | ***P*** |
| --- | --- | --- |
| bladder | 0.153 | 2.5 × 10^-3^ |
| breast | 0.223 | 2.9 × 10^-12^ |
| cervical | 0.204 | 5.4 × 10^-3^ |
| lung^1^ | 0.102 | 0.03 |
| lung^2^ | 0.252 | 7.0 × 10^-4^ |
| head and neck | 0.183 | 4.0 × 10^-5^ |
| stomach | 0.100 | 0.06 |
| pancreas | 0.091 | 0.32 |
| thyroid | 0.040 | 0.37 |
| kidney | 0.031 | 0.61 |

“*” Sample size for each cancer type: *: bladder (388), breast (N = 951), cervical (N = 179), lung adenocarcinoma (N = 471), lung squamous carcinoma (N = 176), head and neck (N = 477), stomach (N = 83), pancreas (N = 99), thyroid (N = 372), and kidney (N = 160). “^1^” and “^2^” refers to lung adenocarcinoma and lung squamous carcinoma, respectively.

**Table S5. Association analyses of APOBEC-mutational signature with isoform expression levels of *APOBEC3A* and *APOBEC3B* stratified by clinical subtypes in breast cancer.**

| **Breast Cancer^*^** | **uc003awn** | | **uc011aoc** | | **uc011awo** | |
| --- | --- | --- | --- | --- | --- | --- |
|  | **Beta** | ***P*** | **Beta** | ***P*** | **Beta** | ***P*** |
| Basal | 0.071 | 0.01 | 0.082 | 0.05 | 0.110 | 4.0 × 10^-4^ |
| Her2 | 0.086 | 0.07 | 0.189 | 0.02 | -0.024 | 0.72 |
| LumA | 0.090 | <1.0 × 10^-4^ | 0.121 | 0.9 × 10^-3^ | 0.041 | 0.18 |
| LumB | -1 × 10^-4^ | 0.99 | 0.069 | 0.20 | 0.062 | 0.16 |

“*” Sample size for each molecular of clinical subtype predicted based on PAM50: Basal (N = 152), Her2 (N = 71), LumA (N = 438) and LumB (N = 179). Multivariate regression analysis was constructed to include all six isoforms as independent variables and APOBEC-signature mutation as the dependent variable for each cancer type.

**Table S6. The distribution of deletion genotypes in samples for each cancer type**

| **Cancer type** | **No deletion** | **Heterozygous deletion** | **Homozygous deletion** |
| --- | --- | --- | --- |
| bladder | 252 | 25 | 9 |
| breast | 658 | 65 | 11 |
| cervical | 117 | 13 | 3 |
| lung^1^ | 333 | 28 | 2 |
| lung^2^ | 74 | 11 | 0 |
| head and neck | 299 | 23 | 1 |
| stomach | 59 | 6 | 1 |
| pancreas | 86 | 4 | 0 |
| thyroid | 284 | 47 | 2 |
| kidney | 125 | 17 | 1 |

“^1^” and “^2^” refers to lung adenocarcinoma and lung squamous carcinoma, respectively.

**Table S7:** A list of top enriched canonical pathways for genes that were co-expressed with the isoform uc011aoc across cancer types.

| **Cancer type** | **Ingenuity Canonical Pathways** | **-log (*P* value)** |
| --- | --- | --- |
| bladder | HIPPO signaling | 2.17 |
|  | Purine Ribonucleosides Degradation to Ribose-1-phosphate | 1.8 |
|  | Role of Oct4 in Mammalian Stem Cell Pluripotency | 1.76 |
|  | Cell Cycle: G2/M DNA Damage Checkpoint Regulation | 1.72 |
|  | Embryonic Stem Cell Differentiation into Cardiac Lineages | 1.58 |
| breast | PTEN Signaling | 3.85 |
|  | Cancer Drug Resistance By Drug Efflux | 2.78 |
|  | Epithelial Adherens Junction Signaling | 2.71 |
|  | Systemic Lupus Erythematosus Signaling | 2.51 |
|  | GM-CSF Signaling | 2.4 |
| cervical | Amyloid Processing | 1.75 |
|  | BMP signaling pathway | 1.42 |
|  | TGF-β Signaling | 1.29 |
|  | Cardiomyocyte Differentiation via BMP Receptors | 1.23 |
|  | Wnt/β-catenin Signaling | 0.879 |
| lung adenocarcinoma | iNOS Signaling | 4.33 |
|  | Interferon Signaling | 3.58 |
|  | IL-10 Signaling | 2.55 |
|  | Parkinson's Signaling | 2.54 |
|  | IL-22 Signaling | 2.44 |
| lung carcinoma | Retinoic acid Mediated Apoptosis Signaling | 1.97 |
|  | Thyronamine and Iodothyronamine Metabolism | 1.86 |
|  | Thyroid Hormone Metabolism I (via Deiodination) | 1.86 |
|  | Pentose Phosphate Pathway (Non-oxidative Branch) | 1.74 |
|  | Pentose Phosphate Pathway | 1.5 |
| head and neck | April Mediated Signaling | 1.95 |
|  | Role of Pattern Recognition Receptors | 1.91 |
|  | B Cell Activating Factor Signaling | 1.9 |
|  | TREM1 Signaling | 1.67 |
|  | Communication between Innate and Adaptive Immune Cells | 1.63 |
| stomach | Acyl-CoA Hydrolysis | 4.97 |
|  | Methylglyoxal Degradation III | 4.82 |
|  | Stearate Biosynthesis I (Animals) | 4.75 |
|  | Dolichyl-diphosphooligosaccharide Biosynthesis | 3.59 |
|  | Bile Acid Biosynthesis, Neutral Pathway | 3.27 |
| pancreas | DNA Double-Strand Break Repair by Recombination | 2.77 |
|  | Fatty Acid α-oxidation | 2.63 |
|  | Remodeling of Epithelial Adherens Junctions | 2.53 |
|  | Actin Nucleation by ARP-WASP Complex | 2.51 |
|  | Role of BRCA1 in DNA Damage Response | 2.47 |
| thyroid | EIF2 Signaling | 2.06 |
|  | Ceramide Biosynthesis | 1.63 |
|  | Zymosterol Biosynthesis | 1.55 |
|  | Cholesterol Biosynthesis I | 1.22 |
|  | Cholesterol Biosynthesis II (via 24,25-dihydrolanosterol) | 1.22 |
| kidney | GPCR-Mediated Integration of Enteroendocrine Signaling | 2.87 |
|  | β-alanine Degradation I | 2.44 |
|  | Lanosterol Biosynthesis | 2.44 |
|  | TNFR1 Signaling | 1.98 |
|  | Methylmalonyl Pathway | 1.97 |

**Table S8:** Associations between predicted neoantigen loads and germline *APOBEC3A/B* deletion.

| **Cancer type^*^** | **Freq** | |
| --- | --- | --- |
|  | **Beta** | ***P*** |
| bladder | 0.270 | 0.16 |
| breast | -0.422 | 6.5 × 10^-3^ |
| cervical | 0.463 | 0.18 |
| lung^1^ | 0.073 | 0.77 |
| lung^2^ | 0.032 | 0.91 |
| head and neck | 0.061 | 0.80 |
| stomach | 0.127 | 0.84 |
| pancreas | -0.544 | 0.38 |
| thyroid | 0.042 | 0.84 |
| kidney | -0.050 | 0.81 |

“*” Sample size for each cancer type: bladder (N = 286), breast (N = 734), cervical (N = 133), lung adenocarcinoma (N = 363), lung squamous carcinoma (N = 85), head and neck (N = 323), stomach (N = 66), pancreas (N = 90), thyroid (N = 333), kidney (N = 143). “^1^” and “^2^” refers to lung adenocarcinoma and lung squamous carcinoma, respectively. The significance level at *P* = 0.005, corresponding to a threshold with a Bonferroni-correction of *P* = 0.05, given 10 tests.

**Table S9:** Associations between predicted neoantigen loads and APOBEC-mutational signature.

| **Cancer type^*^** | **Beta** | ***P*** |
| --- | --- | --- |
| bladder | 0.627 | 1.5 × 10^-90^ |
| breast | 0.615 | 2.1 × 10^-125^ |
| cervical | 0.649 | 6.0 × 10^-49^ |
| lung^1^ | 0.666 | 2.6 × 10^-85^ |
| lung^2^ | 0.447 | 1.6 × 10^-16^ |
| head and neck | 0.526 | 3.9 × 10^-6^ |
| stomach | 1.037 | 1.3 × 10^-13^ |
| pancreas | 0.643 | 3.5 × 10^-14^ |
| thyroid | 0.428 | 2.9 × 10^-10^ |
| kidney | 0.323 | 1.4 × 10^-5^ |

“*” Sample size for each cancer type: *: bladder (388), breast (N = 951), cervical (N = 179), lung adenocarcinoma (N = 471), lung squamous carcinoma (N = 176), head and neck (N = 477), stomach (N = 83), pancreas (N = 99), thyroid (N = 372), and kidney (N = 160). “^1^” and “^2^” refers to lung adenocarcinoma and lung squamous carcinoma, respectively. The significance level at *P* = 0.005, corresponding to a threshold with a Bonferroni-correction of *P* = 0.05, given 10 tests.

**Table S10:** Associations between predicted neoantigen loads and proportion of APOBEC-mutational signature.

| **Cancer type^*^** | **Beta** | ***P*** |
| --- | --- | --- |
| bladder | 4.590 | 8.9 × 10^-29^ |
| breast | 3.558 | 2.8 × 10^-27^ |
| cervical | 4.786 | 1.7 × 10^-15^ |
| lung^1^ | 0.804 | 0.22 |
| lung^2^ | 1.401 | 0.05 |
| head and neck | 2.964 | 4.5 × 10^-14^ |
| stomach | -8.433 | 0.01 |
| pancreas | 7.286 | 0.10 |
| thyroid | 1.619 | 0.03 |
| kidney | 0.161 | 0.89 |

“*” Sample size for each cancer type: *: bladder (388), breast (N = 951), cervical (N = 179), lung adenocarcinoma (N = 471), lung squamous carcinoma (N = 176), head and neck (N = 477), stomach (N = 83), pancreas (N = 99), thyroid (N = 372), and kidney (N = 160). “^1^” and “^2^” refers to lung adenocarcinoma and lung squamous carcinoma, respectively. Univariate regression analysis: Neoantigen loads ~ proportion of APOBEC-mutational signature. The significance level at *P* = 0.005, corresponding to a threshold with a Bonferroni-correction of *P* = 0.05, given 10 tests.

**Table S11:** Associations between abundance of relative immune cell compositions in TILs and neoantigen loads.

| **Cancer type^*^** | **B cell naïve** | | **B cell memory** | | **T.cells.CD8** | | **T.cells.CD4.memory.activated** | |
| --- | --- | --- | --- | --- | --- | --- | --- | --- |
|  | **Beta** | ***P*** | **Beta** | ***P*** | **Beta** | ***P*** | **Beta** | ***P*** |
| bladder | -0.0110 | 9.5 × 10^-4^ | -0.0056 | 0.05 | 0.0133 | 1.1 × 10^-3^ | 0.0028 | 0.02 |
| breast | -0.0040 | 2.4 × 10^-3^ | -0.0021 | 7.3 × 10^-5^ | 0.0002 | 0.90 | 0.0001 | 0.68 |
| cervical | -0.0004 | 0.80 | -0.0006 | 0.45 | 0.0098 | 0.04 | 0.0003 | 0.79 |
| lung^1^ | 0.0002 | 0.82 | -0.0034 | 0.02 | 0.0026 | 0.11 | 0.0016 | 9.8 × 10^-4^ |
| lung^2^ | -0.0062 | 0.02 | -0.0016 | 0.30 | 0.0028 | 0.47 | 0.0022 | 0.17 |
| head and neck | -0.0003 | 0.76 | -0.0020 | 0.07 | 0.0050 | 0.06 | 0.0026 | 4.6 × 10^-4^ |
| stomach | -0.0023 | 0.74 | -0.0092 | 0.18 | 0.0082 | 0.15 | 0.0028 | 0.12 |
| pancreas | -0.0059 | 0.55 | -0.0028 | 0.48 | 0.0024 | 0.50 | 0.0013 | 0.03 |
| thyroid | -0.0065 | 0.09 | -0.0017 | 0.57 | -0.0053 | 0.21 | -0.0004 | 0.06 |
| kidney | -0.0092 | 0.15 | -0.0034 | 0.51 | -0.0211 | 0.18 | 0.0001 | 0.17 |

“*” Sample size for each cancer type: *: bladder (196), breast (N = 595), cervical (N = 131), lung adenocarcinoma (N = 406), lung squamous carcinoma (N = 58), head and neck (N = 423), stomach (N = 163), pancreas (N = 75), thyroid (N = 46), and kidney (N = 117). “^1^” and “^2^” refers to lung adenocarcinoma and lung squamous carcinoma, respectively. The significance level at *P* = 0.005, corresponding to a threshold with a Bonferroni-correction of *P* = 0.05, given 10 tests.

**Table S12:** Associations between abundance of relative immune cell compositions in TILs and APOBEC-mutational signature.

| **Cancer type^*^** | **B cell naïve** | | **B cell memory** | | **T.cells.CD8** | | **T.cells.CD4.memory.activated** | |
| --- | --- | --- | --- | --- | --- | --- | --- | --- |
|  | **Beta** | ***P*** | **Beta** | ***P*** | **Beta** | ***P*** | **Beta** | ***P*** |
| bladder | -0.00647 | 0.02 | -0.00575 | 0.01 | 0.01099 | 9.0 × 10^-4^ | 0.00238 | 0.02 |
| breast | -0.00128 | 0.29 | -0.00124 | 0.01 | 0.00227 | 0.07 | 0.00049 | 0.05 |
| cervical | -0.00076 | 0.51 | -0.00038 | 0.52 | 0.00507 | 0.16 | 0.00018 | 0.85 |
| lung^1^ | 0.00045 | 0.64 | -0.00308 | 0.02 | 0.00266 | 0.07 | 0.00106 | 0.01 |
| lung^2^ | -0.00297 | 0.15 | 0.00041 | 0.74 | 0.00515 | 0.10 | 0.00227 | 0.08 |
| head and neck | 0.00038 | 0.63 | -0.00164 | 0.05 | 0.00344 | 0.09 | 0.00144 | 9.8 × 10^-3^ |
| stomach | 0.00011 | 0.99 | -0.00887 | 0.43 | 0.00732 | 0.43 | 0.00538 | 0.06 |
| pancreas | -0.02636 | 6.1 × 10^-3^ | -0.00399 | 0.31 | -0.00120 | 0.73 | 0.00051 | 0.39 |
| thyroid | -0.00328 | 0.49 | -0.00383 | 0.30 | -0.00514 | 0.33 | -0.00020 | 0.41 |
| kidney | 0.00187 | 0.75 | 0.00686 | 0.13 | -0.00424 | 0.76 | 0.00005 | 0.23 |

“*” Sample size for each cancer type: *: bladder (196), breast (N = 595), cervical (N = 131), lung adenocarcinoma (N = 406), lung squamous carcinoma (N = 58), head and neck (N = 423), stomach (N = 163), pancreas (N = 75), thyroid (N = 46), and kidney (N = 117). “^1^” and “^2^” refers to lung adenocarcinoma and lung squamous carcinoma, respectively. The significance level at *P* = 0.005, corresponding to a threshold with a Bonferroni-correction of *P* = 0.05, given 10 tests.

**Figure S1:** The expression levels of six isoforms of *APOBEC3A* and *ABOBEC3B* for each cancer type.


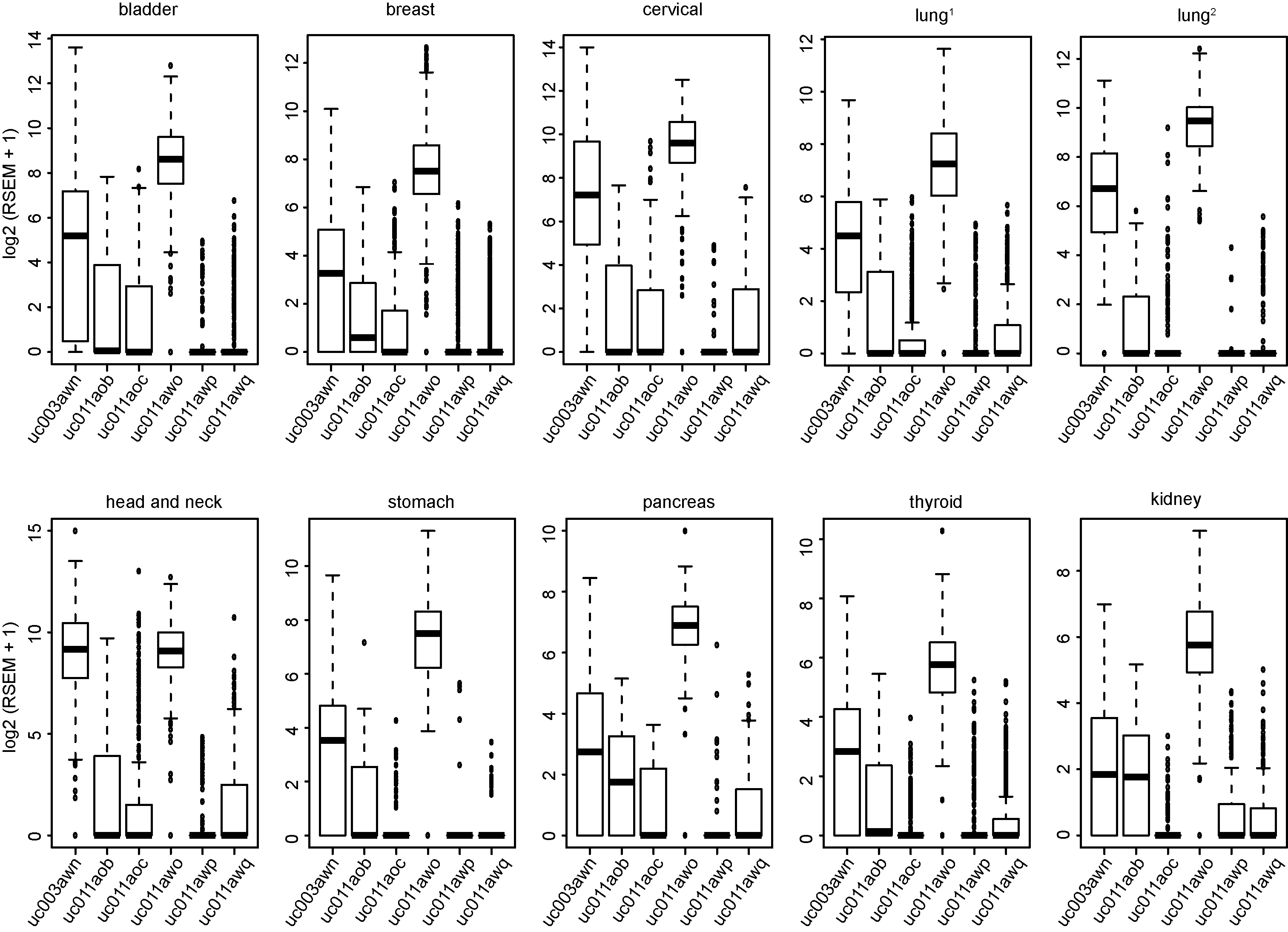

Supplement: Supplementary file 1 — Additional file 1: Table S1. Associations between APOBEC-mutational signature and gene expression levels of APOBEC3A and APOBEC3B. Table S2. Associations between APOBEC-mutational signature and gene expression levels of APOBEC3C, APOBEC3D, APOBEC3F, APOBEC3G, and APOBEC3H. Table S3. Associations between APOBEC-mutational signature and each isoform expression level of APOBEC3A and APOBEC3B. Table S4. Expression correlation between APOBEC3A with the isoform uc011aoc for each cancer types. Table S5. Associations between APOBEC-mutational signature and isoform of APOBEC3A and APOBEC3B stratified by clinical subtypes in breast cancer. Table S6. The distribution of deletion genotypes in samples for each cancer type. Table S7. A list of top enriched canonical pathways for genes that were co-expressed with the isoform uc011aoc across cancer types. Table S8. Associations between predicted neoantigen loads and germline APOBEC3A/B deletion. Table S9. Associations between predicted neoantigen loads and APOBEC-mutational signature. Table S10. Associations between predicted neoantigen loads and proportion of APOBEC-mutational signature. Table S11. Associations between abundance of relative immune cell compositions in TILs and neoantigen loads. Table S12. Associations between abundance of relative immune cell compositions in TILs and APOBEC-mutational signature. Figure S1. The expression levels of six isoforms of APOBEC3A and ABOBEC3B for each cancer type. [file 12920_2019_579_MOESM1_ESM.docx]
